# Asymmetric Information in Iranian’s Health Insurance Market: Testing of Adverse Selection and Moral Hazard

**DOI:** 10.5539/gjhs.v7n6p146

**Published:** 2015-04-15

**Authors:** Farhad Lotfi, Hassan Abolghasem Gorji, Ghadir Mahdavi, Mohammad Hadian

**Affiliations:** 1 Department of Health Economics, School of Health Management and Information Sciences, Iran University of Medical Sciences, Tehran, Iran; 2 Department of Health Services Management, School of Health Management and Information Sciences, Iran University of Medical Sciences, Tehran, Iran; 3 Health Management and Economics Research Center, Iran University of Medical Sciences, Tehran, Iran; 4 ECO College of Insurance, Allameh Tabataba’i University, Tehran, Iran

**Keywords:** adverse selection, asymmetric information, moral hazard

## Abstract

**Background:**

Asymmetric information is one of the most important issues in insurance market which occurred due to inherent characteristics of one of the agents involved in insurance contracts; hence its management requires designing appropriate policies. This phenomenon can lead to the failure of insurance market via its two consequences, namely, adverse selection and moral hazard.

**Objective:**

This study was aimed to evaluate the status of asymmetric information in Iran’s health insurance market with respect to the demand for outpatient services.

**Materials/sPatients and Methods:**

This research is a cross sectional study conducted on households living in Iran. The data of the research was extracted from the information on household’s budget survey collected by the Statistical Center of Iran in 2012. In this study, the Generalized Method of Moment model was used and the status of adverse selection and moral hazard was evaluated through calculating the latent health status of individuals in each insurance category. To analyze the data, Excel, Eviews and stata11 software were used.

**Results:**

The estimation of parameters of the utility function of the demand for outpatient services (visit, medicine, and Para-clinical services) showed that households were more risk averse in the use of outpatient care than other goods and services. After estimating the health status of households based on their health insurance categories, the results showed that rural-insured people had the best health status and people with supplementary insurance had the worst health status. In addition, the comparison of the conditional distribution of latent health status approved the phenomenon of adverse selection in all insurance groups, with the exception of rural insurance. Moreover, calculation of the elasticity of medical expenses to reimbursement rate confirmed the existence of moral hazard phenomenon.

**Conclusions:**

Due to the existence of the phenomena of adverse selection and moral hazard in most of health insurances categories, policymakers need to adjust contracts so that to reduce these phenomena. Given the importance of financing, the presence of such problems can lead to less coverage of health insurance provided by insurers, loss of contracts with health care institutions and service providers, and lower quality of health services.

## 1. Introduction

The phenomenon of asymmetric information due to insured’s extrinsic and inherent properties as one of the agents involved in insurance contracts and requires designing appropriate policies. Lack of ability to recognize Insured people with various risks can pave the way for the development of adverse selection, which is one of the consequences of asymmetric information. Although several mechanisms are used by health Insurance organizations to assess the health status of the population, information asymmetric may still occur. Moral hazard is another clear indication of asymmetric information which occurs when there is a level of uncertainty with regard to some variables such as requesting unnecessary medical services (Resende & Zeidan, 2010).

The topics of asymmetric information, adverse selection, and moral hazard in the insurance literature are rooted in researches done by Arrow, Pauli and Rothschild et al. These subjects were first proposed by the economists (Arrow, 1963; Pauly, 1974; Rothschild & Stiglitz, 1976).

The models of adverse selection and moral hazard indicate the positive relationship between risk and demand for insurance (Doiron, Jones, & Savage, 2008). Adverse selection will emerge when insured people have some information about their risks which are not known by insurers; in such cases insured people use this type of information to buy insurance (Cohen & Siegelman, 2010).

Most of the theoretical works in the field of insurance show that asymmetric information lead to the provision of inadequate insurance services or even the failure of the insurance market. In the traditional theory of demand for services, there are several hypotheses that lead to adverse selection that one of them is the existence of asymmetric information (Gh Mahdavi & Izadi, 2012; Ghadir Mahdavi & Rinaz, 2006). Its’ outcomes can potentially increase the use of medical services, affect on consumption behaviors of individuals, and ultimately increase health expenditure (Abolhallaje et al., 2013).

During the last decade, the growth of inflation in health care costs in the United States of America has been more than the consumer price index and a high percentage of its Gross domestic product (GDP) has been devoted to health expenditure; some researchers believe that such an inflation is rooted in the deviations in health care Insurance market (Bajari, Hong, & Khwaja, 2006). During the same period, Iran has also experienced a growth in health expenses compared with other consumer costs (Haddad & Anbaji, 2010).

The lack of sufficient information about the health status and heavy burden of medical expenses are part of the reasons for this deviation in health insurance market. The lack of information on the Latent health status of population leads to adverse selection; moreover, the burden of medical expenses is due to the phenomenon of moral hazard (Liu, Nestic, & Vukina, 2012).

Given the fact that the insured people do not directly pay for all medical costs while receiving medical care services, a high share of medical costs is paid by Insurance institutions. Therefore they don’t have any motivation to limit their consumption and will cause inflation in health expenditure (Cutler & Zeckhauser, 2000).

More than half of the insured people who are under the coverage of employer sponsored insurances in the USA, have the chance to choose plans that are different in terms of generosity from among competing plans for health Insurance (Tchernis, Normand, Pakes, Gaccione & Newhouse, 2006).

Even though the competition between the various insurance institutions can reduce the insurance costs and can fulfill heterogeneous needs of consumers, it can exacerbate adverse selection. Usually people with more severe health conditions choose plans that are more generous. This situation leads to welfare losses resulting from the financial inability of insurance companies against future risks, and it may even limit insurance contracts while people demand to buy insurance (Rothschild & Stiglitz, 1976).

In addition, determining similar and uniform premium fee independent of risk in insurance plans for health services is accepted as a part of “managed competition”; it is not consistent with the condition of optimality in insurance contracts at the presence of moral hazard; hence risk-based premiums should be considered as an alternative (Zweifel & Breuer, 2006).

### 1.1 Relationship to the Literature

Different studies have been conducted on the adverse selection and moral hazard in different countries. Bajari et al. used a semi-parametric approach to address these issues in different insurance groups in the USA; their results showed that individuals under the coverage of Medicare insurance had worse health status than other insured people, and the lowest risk was related to persons with self-employed insurance. Moreover, the distribution of health status among the uninsured people was significantly different from other people under the coverage of different classes of insurance. Further moral hazard test showed that the people covered by Medicare had no significant difference with the uninsured people; it shows that this type of insurance had better reimbursement plans which improved the consumption behavior of insured people under its’ coverage (Bajari et al., 2006).

Mahdavi et al. assessed the status of adverse selection in supplementary insurance markets in Tehran (Iran). They used two logistic regression models to assess the impact of individuals’ characteristics on the purchase of supplementary insurance plans; finally, because of the relationship between the claim occurrence and the decision to buy insurance, they proved the existence of adverse selection in the market (Gh Mahdavi & Izadi, 2012).

In the same way, Marquis and Philips also evaluated the adverse selection in health supplementary insurance markets. Using probit regression model, they examined the effects of the price of insurance, expected medical expenses, and other factors on the decision to buy supplementary insurance plans. Their results showed a considerable demand for supplementary insurance plans even after the elimination of the current subsides which usually leads to a decrease in demand (Marquis & Phelps, 1987).

Moreover, Kang et al. assessed the effect of private health insurance on medical visits in Korea using a probit model; the results showed that people with private health insurance were 14 percent more likely to refer to physicians than those without this type of insurance. They believed, private insurance in Korea had the potential to create moral hazard (Kang, You, Kwon & Oh, 2009).

Pylypchuk studied the effect of health insurance on the use of prescribed drugs in different types of patients and the results showed that, in comparison with uninsured people and those who were insured but did not have a coverage for drugs, insured persons who had drug coverage in their Insurance plan were more prone to use more medication and medical services than (Pylypchuk, 2010).

Despite the results of the above mentioned studies, the results of a study by Cardon et al. conducted on the effects of adverse selection on the costs and utilization of medical services in the health insurance market, showed no evidence of adverse selection in health insurance markets (Cardon & Hendel, 2001).

Paccagnella studied the voluntary private health insurance among people aged 50 years old in 11 European countries and showed that insurers had been able to control moral hazard and adverse selection by taking into account the cost- sharing for every individuals; this led to a situation in which the people in all of these countries, except for the Netherlands, paid more out of pocket payment (Paccagnella, Rebba, & Weber, 2013).

By measuring moral hazard and adverse selection in Hong Kong, Wong found no evidences for the excessive demand for services among those who self purchased insurance plans. Hence, the assumption of the existence of adverse selection in the use of public hospital services for this group of people was rejected (Wong et al., 2010).

### 1.2 The Iranian Health Insurance Market

In Iran, many organizations provide health insurance services both in two forms of basic and supplementary insurance plans. In the field of basic insurance, four main institutions are in charge of the provision of such basic services. In 2012, about half of the population of the country was under the coverage of insurance services provided by the Social Security Organization (SSO) which was the largest share among all the involved organizations. Generally, people who are covered by this insurance organization have either compulsory or voluntary insurance. The Medical Service Insurance Organization (MSIO) is another insurance organization that widely provides coverage for a large population in Iran. This organization is in charge of providing insurance coverage for government employees, rural people, students, and other segments of the community. The Military Personnel Insurance Organization provides the insurance services for armed forces and their families. Imam Khomeini Relief Foundation (IKRF) is also another institution which covers poor people, people with disabilities, and orphans; compared with the three above mentioned entities it covers a smaller population. In addition to these institutions, there are several insurance funds which provide their Insurance services only to their own employees and their families (Abolhallaje & Tahereh, 2013; Davari, Haycox & Walley, 2012).

In Iran, people are forced to choose basic insurance based on their occupation and they have no choice to choose their basic insurance; they can only accept or refuse to register for a supplementary insurance plan. Hence, given the importance of health Insurance plans, this study was aimed to evaluate the status of asymmetric information in Iran’s health insurance market via testing its two main outcomes i.e. adverse selection and moral hazard.

## 2. Materials and Methods

This research is a cross sectional study conducted on households living in Iran. It was carried out via the following steps.

### 2.1 First Step: Specifying and Estimating the Utility Function

Given the fact that, every individual manages its own medical consumption on the basis of the type of insurance and information about its own health status, hence a level of medical services is consumed whose utility is maximized regarding to health status and the use of a composite commodity. Like Bajari et al. first we specified the consumer utility function as *U(c,m−θ;γ)*, where *c* is the household consumption of composite commodity, *m* is the consumption of medical services, θ is the latent health status of the individual that is invisible to insurer, and γ is utility function parameter (Bajari et al., 2006). Therefore, the use of medical services can be considered as a substitute for the poor health condition (Cardon & Hendel, 2001). In this function, *m* and θ are assumed to be in the form of monetary units and only their difference is entered in the utility function. Finally, the above mentioned utility function is considered as a separable function with the following form; it also shows the budget constraint that each household faces to.


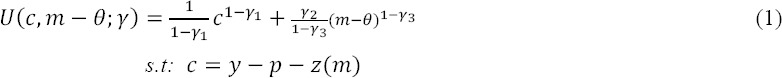


The above utility function also shows the parameters of risk aversion for the consumption of composite commodity and medical services. In the above mentioned budget constraint, *y* represents the total household expenditures, *p* is the premium, and *z(m)* is out of pocket payments spent by households. Hence, insured households pay only part of the total costs and *m-z(m*) reimbursed by insurance institutions. Given that the reimbursed plans by insurance companies do not relate directly to the latent health status of an individual and is only a function of the amount of the consumption of medical services (*m*), this leads to moral hazard. Therefore, individuals consume a level of medical services which maximizes their utility with respect to its own health. Accordingly, as the health status becomes worse, there would be higher tendency to use more medical services. As a result, first order condition is as follows:


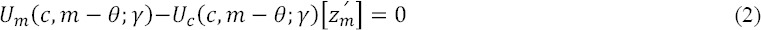


In the first equation, based on the marginal rate of substitution law, people choose a level of medical services which maximize the utility on the basis of their current insurance status. This low is based on the allocation of budget between medical services and composite commodity and it states that the ratio of marginal utility of both commodities with respect to the relative prices should be equal. Hence the optimal condition is as follows and the equation is used to estimate the model:


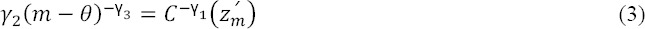


Where, zm′ is the rate of out of pocket payments that the rate for outpatients’ services is equal to 1 for uninsured people and 0.3 for insured people in all insurance categories in Iran. Therefore:


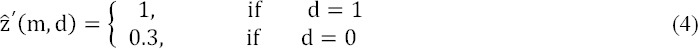


Where d = 1 is representative of uninsured people and d = 0 indicated different insured groups. Given the values for different parameters of utility function for each individual, latent health status can be obtained via calculating consumption of medical services by using optimality condition in Equation 3. Therefore:


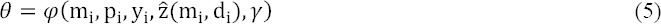


And the explicit form of this function, assuming that m≥0, is as follows:


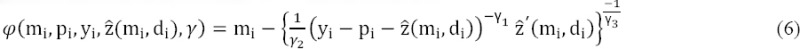


### 2.2 Second Step: Estimating the Model

Firstly, using a semi-parametric approach, the parameters of utility function (*γ*) were estimated. For this estimation, generalized method of movement model was used. As a positive feature of this model, it does not need to apply the parametric assumptions about the distribution of the parameters. Thus, latent health status can be determined through using optimality conditions in Equation 3. Also, the risk aversion parameters of the utility function can be estimated. Thus, it is very important to correctly specify the consumer utility function and utilize the economic assumption of maximum utility. The method also uses a series of instrumental variables that are independent of the individuals’ latent health status and only show the exogenous variations among different health insurance plans.

### 2.3 Adverse Selection and Moral Hazard Test

Following bajari et al. to conduct adverse selection test, we used the conditional distribution of the latent health status in each of the classes of insurance with respect to the distribution of latent health status. Using Kolmogorov-Smirnov test, the health status of individuals at different insurance classes was compared.

To perform moral hazard test, we used the estimated parameters of the utility function and the distribution of latent health status to estimate the elasticity of total medical expenses with regard to the amount of reimbursement. The calculated elasticity shows the percentage of changes in the demand for medical services with regard to the percentage of changes in reimbursed rate by Insurance institutions. This method of moral hazard measurement reflects the changes in consumer behavior with respect to the change in reimbursement policies. Therefore, the distribution of elasticity for each household is calculated by the following equation.


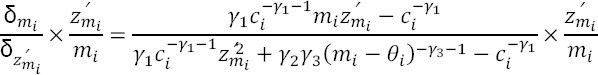


### 2.4 Data

The required data was extracted from household budget questionnaire run by the Statistical Center of Iran in 2012. Each observation shows the information about the amount of out-patients services including visits, Para-clinical services, and medicine consumption. The sample consisted of 34,023 households. Our focus was on the types of insurance. The data consisted of a set of socio-economic variables (such as age, gender, education, household size, income deciles, income and expenses), information about the insurance (including premium, insurance type), and finally the variables related to the utilization of outpatient services (OOP payments by households for outpatient services and amount of reimbursement paid by institutions under contract). Excel software was used to refine the data and then stata11 and Eviews software were used to estimate model parameters.

### 2.5 Inclusion and Exclusion Criteria

The questionnaires which satisfied the requirements of the budget constraint in the utility function were entered into the analysis process and the rest of data was excluded from the study. Taking into account the budget constraint, 57 cases which were not satisfied the budget constraint function were excluded and 33966 households were analyzed.

## 3. Results

The distribution of data showed that, of the 33,966 households that were analyzed, 9,464 were insured by the Social Security Organization (SSO) of whom a total of 5,444 had compulsory employee-employer insurance and the rest were self-insured (voluntary). In addition, 11,843 households were under the coverage of rural insurance; moreover, 3,707 households were officially-insured that both were covered by The Medical Service Insurance Organization (MSIO). A total of 1892 households had supplementary insurance and 511 households covered by other public institutions. Also 6549 were not covered by any type of insurance.

The result of estimating the parameters of the utility function is shown in Table 1. As the t-statistic shows, all these parameters are statistically significant at the 0.01 level. Also, j-statistics indicates instrumental variables including household size, number of literate family members, and the premium met our expectations regarding the parameters of the utility function. These instrument variables haven’t any correlation with latent health status and only reflect exogenous changes. Moreover, the assumption of normality of the data was confirmed using normality test. The risk aversion index γ_3_ was 0.767 for health care consumption (visits, medicine, and Para-clinical services) and γ_1_ was 0.037 for the composite commodities which suggest that people are more risk averse in utilizing medical goods than composite commodities. Moreover, the value of γ_2_ also shows individuals put more emphasis and weight on the consumption of medical services.

**Table 1:** Estimation of the parameters of the utility function (risk aversion parameters for the utilization of medical services and composite commodities) using generalized method of movement

Characteristics	γ_1_	γ_2_	γ_3_
Value	0.037157	73.58	0.767806
t-Statistic	12.58375	3.065228	80.66861
Prob.	0.0000	0.0022	0.0000

J-statistic: 2.11E-05, R-squared, 0.141281.

In addition, Arro-Prat risk aversion index was calculated for different income categories; Table 2 shows the level of this index for different income categories. As the table shows, the higher the amount of income has the lower the degree of risk aversion.

**Table 2:** Arro-Prat risk aversion based on the income category

Income group	Average	Minimum	Maximum
Docile 1	1.38E-06	6.27E-07	3.97E-06
Docile 2	1.35E-06	6.26E-07	3.74E-06
Docile 3	1.33E-06	6.22E-07	3.71E-06
Docile 4	1.32E-06	6.15E-07	3.91E-06
Docile 5	1.23E-06	6.15E-07	3.58E-06
Docile 6	1.12E-06	5.97E-07	3.55E-06
Docile 7	1.15E-06	6.07E-07	3.59E-06
Docile 8	1.07E-06	6.09E-07	3.51E-06
Docile9	1.07E-06	6.09E-07	3.87E-06
Docile 10	9.44E-07	5.84E-07	3.52E-06

After estimating the parameters of the utility function, the latent health status was calculated (using Equation 4). Overall, the mean latent health status was 87 114.72 Iran Rail (Each US dollars equals 12260 Iran Rails in 2012) and the results showed that, from the perspective of insurance institutions, rural-insured’s households had the best health status and individuals with supplementary insurance had the worst health status. The health status in terms of monetary units (Iran Rail) is presented in Table 4 for all the households based on their insurance categories.

**Table 3:** Distribution of health status among different insurance classes and uninsured people

Insurance category	Covered by	Mean	Minimum	Maximum
Uninsured	-	64 968.96	-261 769	26 765 601
Employee-employer insurance	SSO	100 400.4	-1 254 833	1.51E+08
Self-insured insurance		280 782.3	-1 263 548	82 263 195
Officially- insured insurance	MISO	273 618.9	-1 260 580	1.15E+08
Rural insurance		-161 737	-1 295 434	56 818 300
Other public institution insurance	Other institution	322 953.7	-1 227 236	1.59E+08
Supplementary insurance	Public & private Co.	842638	-1252285	2.19E+08

**Table 4:** Results of the distribution of latent health status calculated by Kolmogorov-Smirnov test

Insurance category	1	2	3	4	5	6
0	0.662(0.000)	0.5985(0.000)	0.6285(0.000)	0.6923(0.000)	0.6753(0.000)	0.5455(0.000)
1		0.0823(0.000)	0.0391(0.000)	0.1487(0.000)	0.2572(0.000)	0.1262(0.000)
2			0.0769 (0.000)	0.0983 (0.000)	0.1759 (0.000)	0.0790 (0.000)
3				0.1523(0.000)	0.2394 (0.000)	0.0921 (0.000)
4					0.1198 (0.000)	0.1635 (0.000)
5						0.2009 (0.000)

Insurance categories: 0= uninsured, 1= Employee-employer, 2=Self-insured, 3=officially- insured, 4= Rural-insured, 5= other public institution, 6= Supplementary.

To examine the two outcomes of asymmetric information i.e. adverse selection and moral hazard we used the conditional distribution test of Kolmogorov-Smirnov. To evaluate the status of adverse selection, we calculated the latent health status of the household to test the null hypothesis which suggested the equal distribution of households’ latent health status among different insurance classes. The results of the comparison for different classes of insurance are shown in Table 4. Considering the test statistic, the null hypothesis, which suggested the equal distribution between the uninsured people and the different insurance classes, was rejected; it showed that there is adverse selection in health insurance markets is Iran.

Moreover, Table 5 presents the price elasticity of demand for medical services with regard to the amount of out of pocket payments among different insurance classes. As indicated by the mean elasticity values, among different insurance classes, the minimum and maximum amount of elasticity were observed in people with supplementary insurance and people insured by other public institutions, respectively.

**Table 5:** Elasticity index classified by different insurance category and uninsured people

Insurance category	Mean	Minimum	Maximum
Uninsured	-2.45862	-81.886933	-0.02716089
Employee-employer insurance	-3.49835	-154.84001	-0.01571567
Self-insured insurance	-3.04765	-120.56442	-0.04041747
Officially- insured insurance	-3.43166	-228.32415	-0.01624222
Rural insurance	-3.77396	-115.41573	-0.03899935
Other public institution insurance	-3.88417	-97.135432	-0.04054852
Supplementary insurance	-2.74799	-93.808648	-0.00899122

The results of Kolmogorov-Smirnov test, which was conducted to check the conditional distribution of elasticity, showed that the phenomenon of moral hazard was observed in all insured individuals; the severity of moral hazard in people who were insured by other public institutions was more than the other groups. Therefore, with regard to Kolmogorov-Smirnov test and the comparing of elasticity value between uninsured people with insured people in each category, we can find moral hazard in Iran insurance market. Also, the intensity of this phenomenon in different insurance categories has been showed in Table 6.

**Table 6:** The distribution results of elasticity in different insurance category Kolmogorov-Smirnov test

Insurance category	1	2	3	4	5	6
0	0.1265(0.000)	0.0855(0.000)	0.1013(0.000)	0.1843(0.000)	0.24(0.000)	0.0864(0.000)
1		0.0516(0.001)	0.0436(0.012)	0.0919(0.000)	0.1205(0.000)	0.1229(0.000)
2			0.0347(0.103)	0.1113(0.000)	0.1658(0.000)	0.1132(0.000)
3				0.1202(0.000)	0.1528(0.000)	0.0943(0.000)
4					0.0685(0.079)	0.2025(0.000)
5						0.2353(0.000)

Insurance categories: 0= uninsured, 1= Employee-employer, 2=Self-insured, 3=officially- insured, 4= Rural-insured, 5= other public institution, 6= Supplementary.

## 4. Discussion

Asymmetric Information is one of the major issues of insurance. This phenomenon, which is marked by its two outcomes i.e. moral hazard and adverse selection, can greatly influence inflation and rise health care costs (Bajari et al., 2006; Haddad & Anbaji, 2010; Liu et al., 2012). Neglecting this problem can lead to the bankruptcy of insurance organizations and push them out of the market.

In this study, despite the heterogeneity in households’ health status, a semi- parametric approach was used to estimate the parameters of the utility function of the demand for outpatient services. As *γ*_1_ and *γ*_3_ parameters of utility function showed, household are more risk averse in the consumption of medical services compared with other goods and services and they are more inclined to consume more of these services; it is consistent with the results of studies by Bajari et al. and Keshavarz. Also, *γ*_2_ that represents the significance and weight of medical services consumption is in line with the results of the mentioned studies. However, its values are much higher than that of previous studies which indicates that in recent years, people has paid more attention to the consumption of medical services. Common to all these studies, households put more emphasis on the use of medical services. Consistent with other studies, the Arro-Part risk aversion index showed that people with higher incomes are less risk averse than other people.

Households’ health status also showed that households covered by rural insurance - although paid nothing premium and their premiums were paid by the government- had better health status; and, people who were under the coverage of supplementary insurance had the worst health status. Nevertheless, Bajari’s study in the USA showed that self-insured people had better health status and individuals covered by Medicare who government paid for their health insurance had the worst health status. A study by Keshavarz, which was conducted in 2010, showed that employee- employer insured and officially-insured households had the best health status (Bajari et al., 2006; Haddad & Anbaji, 2010).

The results of conditional Kolmogorov-Smirnov test showed that, contrary to uninsured people, all insurance classes had a significant difference in terms of latent health status; thus the presence of adverse selection phenomenon was proved in all groups, except for the rural insured individuals.

But it is obvious that the intensity of this phenomenon varies in different classes of insurance. This outcome is less observed in employee-employer insured people who are covered by the Social Security Organization (SSO) whose insurance is compulsory. On the other hand, the severity of adverse selection was higher among the self-insured people covered by this organization (SSO), which suggests that this insurance category has worse health status in comparison with the compulsory insured people. This point indicates they have more information about their health status than their insurer, and they apply for insurance when alarmed so that to use the insurance benefits. Hence, to prevent adverse selection, the Social Security Organization should utilize more appropriate mechanisms to insure the people who are voluntarily trying to buy insurance. Moreover, other insurance organizations and public institutions regarding to health status of their insured people, must utilize the appropriate screening mechanisms to focus on and evaluate the risk status of insured people and they have to identify high risk individuals.

According to the results, people who had supplementary insurance had showed the highest level of adverse selection; the result is consistent with that of Mahdavi et al.’s study (Gh Mahdavi & Izadi, 2012). Although various insurance companies provide supplementary insurance for groups of people, the individuals are not forced to choose this type of insurance. Thus, according to the results, people who chose this type of insurance had worse health status, compared with the other insured groups.

To test moral hazard, price elasticity was compared in couples of insurance groups. According to the results of Kolmogorov-Smirnov test, this phenomenon was confirmed and the null hypothesis suggesting the absence of this phenomenon was rejected. The result was in line with the results of Kang, Marquis and Pylypchuk that showed people who have health insurance are more sensitive to changes in the amount of reimbursable by the insurance institutions (Kang et al., 2009; Marquis & Phelps, 1987; Pylypchuk, 2010). Due to the elasticity of medical expenses to the reimbursed rate, if the reimbursed rate reduces by insurance institutions, the level of moral hazard among insured people will decrease more intensely and the insured people will have to take more precaution activities. Although people with supplementary insurance had the worst health status among the other insured groups, they had less intense moral hazard than the other insured households. It suggests that the mentioned insurance institution had a better mechanism for controlling and changing the consumption behaviors.

## 5. Conclusion

The presence of these two phenomena in the health insurance market leads to the provision of inadequate health services, lack of adequate coverage of services, and delay in reimbursements by insurers. It will result in the dissatisfaction of insured, insurer and providers. So, considering these outcomes in health insurance market, insurance organizations should be informed about the risk status of their insured people via more exact screening tests. Also, it is necessary for actuaries to determine the appropriate premium for reimbursement of unexpected loss. Then, they have to make policy and enact insurance laws like deductible to oblige insured people to take more precautionary activities and change their consumption behavior so that they control the high burden of medical expenses resulting from moral hazard.
